# Simultaneous Modification of Properties Relevant to the Processing and Application of Virgin and Post-Consumer Polypropylene

**DOI:** 10.3390/polym15071717

**Published:** 2023-03-30

**Authors:** Ines Traxler, Hannes Kaineder, Joerg Fischer

**Affiliations:** 1 Competence Center CHASE GmbH, Altenberger Strasse 69, 4040 Linz, Austria; 2 Institute of Polymeric Materials and Testing, Johannes Kepler University, Altenberger Strasse 69, 4040 Linz, Austria

**Keywords:** polypropylene recyclate, mixing rules, polymer blends, MFR, mechanical properties, design from recycling

## Abstract

Post-consumer recyclates often have a property profile that results from mixing a variety of products, which are made from different materials, produced by different processing methods, and coming from applications with different lifetimes. This usually leads to a mixture of all these material properties in the recycling process. In contrast, virgin materials are specifically designed for applications and thus offer all the necessary properties for the intended products. In order to be able to use recycled materials for specific and demanding applications, not only the viscosity, which is important for processing and often varies greatly with recyclates, but also the mechanical properties, particularly the tensile modulus and impact strength, must be adjusted. For this purpose, various virgin materials of polypropylene homopolymers, random copolymers, and block copolymers with different flowabilities were mixed in different proportions and their properties were determined. The flowability of homopolymers and random copolymers in the blend behaved very similarly, while block copolymers exhibited a different behavior in some cases. By incorporating homopolymers into blends, the stiffness of the resulting material blend can be very well adjusted. The addition of random copolymers can increase strain at break, and the addition of block copolymers results in a significant increase in impact strength. In numbers, the maximum adjustment range for tensile modulus, yield stress, strain at break, and impact strength are 880 MPa, 14 MPa, 185%, and 6.9 kJ/m^2^, respectively. While a good and reliable prediction of property profile is possible for polymer blends with different virgin materials, the resulting material properties for polymer blends of virgin and recycled materials are also influenced by impurities. In this work, however, a good prediction was also achieved for recyclate blends.

## 1. Introduction

Recycling post-consumer plastics offers various possibilities to prevent improper disposal of plastic waste in the environment. In Europe, recycling is regulated on the legal level in the directive 2018/852 of the European Union which states the number of recyclates necessary for packaging products until 2025 and 2030, respectively [1]. However, no specific products are defined yet for the uptake of polyolefin recyclates as it is already regulated for PET bottles [2]. Therefore, several applications need to be found where either modified or unmodified recyclates can be used [3,4,5,6,7].

With a demand for polypropylene (PP) of around 10 Mt in 2019, PP is classified as the second most important material for packaging after polyethylene low-density and polyethylene liner-low-density (PE-LD/PE-LLD) [8]. The high share of PP in packaging products is justified by the low price and the extremely high variation of properties which are provided by the polymer producers. Grades with a low melt flow rate (MFR) are usually used for blow molding and extrusion applications, while high MFR grades are necessary for injection molding. Depending on the product and its demanded properties, either homopolymers (PP-H), random copolymers (PP-R), or block copolymers (PP-B) are used as they all offer individual property profiles. Homopolymers are polymerized using only propylene molecules which leads to high stiffness. Random and block copolymers have incorporated ethylene molecules or higher olefins in the PP backbone either statistically/randomly or in blocks, respectively, which leads to high ductility and high-impact resistance [9].

For products made from PP, individual property profiles with target values for crucial properties are defined. Often, these properties cannot be met by recyclates without further modification [3]. In Table 1, a comparison of different processing methods for PP, as well as the used PP types, ranges of the MFR value, typical properties, and exemplary products are shown. Most recyclates which are currently available on the market are mixtures of different PP products and thus of different PP types such as PP-H, PP-R, and PP-B. Moreover, depending on the depth of sorting, PP recyclates also contain foreign polymers such as polyethylene, which leads to issues of miscibility. Several studies have already discussed the property profile of commercial recyclates [10,11], which usually provide high melting temperatures (above 160 °C), certain amounts of remaining inorganic fillers, rather high MFR values, low stiffness, low ductility due to residual macroscopic contaminants, and impact strengths in the range of virgin materials for notched specimens. A further issue in PP recycling is the thermo-oxidative and UV-light-induced material degradation, causing chain scission. Various studies in laboratory environments were already carried out to address this topic. The main results are an increasing MFR, a decreasing ductility, and a drop in the oxidation induction temperature, which corresponds to a decreased molecular weight [12,13,14,15,16,17].

**Table 1:** Overview of typical types (i.e., H for homopolymers, R for random copolymers, and B for block copolymers) of PP and their corresponding MFR ranges (given in g/10 min) for four different processing methods together with properties required for specific packaging applications. Data are taken from [18,19,20,21].

Process	PP Type	MFR	Necessary Properties	Exemplary Products
Blow molding	R, B	1.3–20	High ductility and impact strength	Bottles and containers
Film extrusion	H, R, B	0.85–13	Good optical properties, high toughness/stiffness	Blown and cast films
Thermoforming	H, R, B	1.2–5	High stiffness, good melt stability	Cups, trays, and blisters
(Injection) molding	H, R, B	1.3–100	Depending on application	Thin wall packaging, caps, and closures

As commercially available recyclates typically have a very unspecific property profile, there is the need for property modification to adjust its property profile for a certain application. While the applicability of the mixing rules for viscosity and MFR adjustments was already widely discussed in the literature [22,23,24,25,26,27,28,29,30] and is already published in a previous study [31], a simultaneous adaption of flowability and mechanical properties was mostly neglected [32]. Especially stiffness (e.g., tensile modulus) and impact behavior (e.g., Charpy notched impact strength) are important parameters, which are necessary for material selection in the design process of a new product. Therefore, these properties are usually stated in data sheets of commercial material grades.

For this study, several different PP grades were selected. Two and three types with similar MFR in the range of pipe (<1 g/10 min) and extrusion grades (8 g/10 min), respectively, were mixed with up to four pre-selected homopolymer grades (8–25 g/10 min) in 20% increments between 0% and 100% via compounding. Other property modifiers such as fillers and compatibilizers were neglected in this study as these components would not be compliant with packaging applications in a circular economy [33]. Since the MFR is an often-used parameter for materials and it is relevant for the processing method, MFR values were determined for each blend and the applicability of mixing rules (i.e., linear, Arrhenius, and Cragoe) was tested. Therefore, MFR measurements instead of rheological measurements are chosen for this research. Nevertheless, both methods reveal information on the chain length of polypropylene grades [10,11]. To investigate application-relevant properties, injection molding of specimens and subsequent determination of mechanical properties were carried out for selected blends. To check potential differences in the adaptability of mechanical properties of recyclates compared to virgin materials, in addition to a blend with a virgin homopolymer, a blend with a post-consumer recyclate (PCR) grade was investigated.

## 2. Materials and Methods

### 2.1. Materials

Nine different virgin PP grades and one PCR grade were used for this study. The materials can be classified as homopolymers, random copolymers, and block copolymers, and this is indicated by the first letter of the material names, namely, H, R, and B, respectively. All materials were delivered by Borealis (Borealis AG, Vienna, Austria) or their subsidiary company mtm plastics (mtm plastic GmbH, Niedergebra, Germany) in case of the recyclate. According to the datasheet, the recyclate originates from pre-sorted municipality waste. A detailed description of the materials, including their MFR values, is given in Table 2. The MFR values were taken from the data sheets and measured at a temperature of 230 °C and with a weight of 2.16 kg. The sample designation is a combination of the type of PP (H, R, or B) and the corresponding MFR according to the datasheet. The recyclate is abbreviated with PCR and the MFR of the used grade. In general, grades with no or minor stabilization were used. Only BD310MO is additivated with antistatic and demolding additives as no other grade with the required MFR was available. Copolymers are PP-based with a certain amount of ethylene phases.

### 2.2. Sample Preparation

For this mixing study, nineteen binary blends divided into three sample sets were produced via compounding. In Table 3, a detailed description of the produced blends is shown. In Sample Set 1, the adaptability of the MFR is evaluated with two pipe grades with rather low MFR values below 1 g/10 min where one is a homopolymer (H0.8) and one is a block copolymer (B0.25). Sample set 2 deals with the impact of different PP types (H8, R8, and B8) with a constant MFR of 8 g/10 min. Finally, Sample Set 3 compares the impact of a homopolymer blending partner on the flowability and mechanical behavior of a virgin material and a post-consumer recyclate (PCR) with similar MFR values. Materials from sample sets 2 and 3 were also injection molded to specimens except for blends containing H12. As indicated in Table 4, compounds of all blends and injection molded specimens of selected blends were made in increments of 20%. The abbreviations $x_{1}$ and $x_{2}$ are related to the weight fraction of material 1 and material 2, respectively.

For the compounding and mixing step, a co-rotating twin-screw extruder of the type ZSE 18 MAXX (Leistritz Extrusionstechnik GmbH, Nuremberg, Germany) was used. The used temperature profile and settings are given in Table 5. After the die, the strand was cooled in a cold-water bath and granulated using a strand pelletizer. For samples where only MFR measurements are required, 50 g of granules were produced for subsequent measurements, and for samples which were selected for injection molding, around 750 g were compounded.

Type 1A multipurpose [34] and Type 1 Charpy specimens [35] for mechanical testing were produced with a Victory 60 injection molding machine (Engel Austria GmbH, Schwertberg, Austria) according to ISO 294 [36]. For multipurpose specimens, a cavity with two specimens, and Charpy specimens, a cavity with four specimens were used. The tool temperature was set to 40 °C. The first few shots were thrown away to ensure constant processing conditions.

### 2.3. Characterization Methods

For the determination of the MFR, an Aflow plastometer (Zwick Roell GmbH & Co. KG, Ulm, Germany) was used. The measurements were carried out according to ISO 1133-1 using the standard test condition of PP which is a temperature of 230 °C and a static load of 2.16 kg [37]. First, around 4 g of granules were filled in the cylinder and compressed to remove air between the granules. Then, a pre-heating period of 300 s was executed. During the measurement itself, six extrudates were produced with a cut after a piston movement of 5 mm. To calculate the MFR, all six extrudates were weighed on a Quintix laboratory scale (Sartorius AG, Goettingen, Germany) exactly to four decimal places to calculate an average value. One measurement per sample was carried out for Sample Set 1 because one measurement of low MFR materials is time-consuming. Nevertheless, it is already an average value of six extrudates. Two measurements were carried out for sample sets 2 and 3.

Tensile testing was carried out using a Z005 universal testing machine (Zwick Roell GmbH & Co. KG, Ulm, Germany) and tactile strain gauges of the type MultiXtens II HP at ambient conditions of 23 °C and relative humidity of 50% at which the specimens were stored for at least 96 h prior testing. Following the standard ISO 527-2 [34], an initial clamping length for 1A multipurpose specimens of 115 mm and a testing speed of 50 mm/min were chosen. In the strain range between 0.05 and 0.25%, a testing speed of 1 mm/min was set for the determination of the tensile modulus. Tensile modulus, yield stress, and strain at break were evaluated. Five specimens were measured per sample.

The Charpy notched impact strength (NIS) was measured using a HIT25P impact tester (Zwick Roell GmbH & Co. KG, Ulm, Germany) according to ISO 179-1 [35]. Therefore, ten specimens per sample were notched on an RM2265 microtome (Leica Biosystems Nussloch GmbH, Nussloch, Germany) with a Type A notch to obtain a remaining width of 8 mm. A pendulum with 0.5 J was used to stay in the proposed absorbed energy range of 10 to 80% of the pendulum as suggested in the standard. The specimens were tested edgewise at a temperature of 23 °C and a relative humidity of 50%.

### 2.4. Mixing Rules

As evaluated in [31], mixing rules according to Arrhenius and Cragoe have been evaluated as applicable to binary PP blends of both virgin and recyclate blends. These mixing rules are given in Table 6. The linear mixing rule is also added as a reference. $MFR_{1}$ and $MFR_{2}$ are the MFR values of both mixing partners given in g/10 min. The parameters $x_{1}$ and $x_{2}$ are the respective weight fractions of both components in the blends and must sum up to 1. The parameter L in the model of Cragoe is set to 2000, as explained in [23,31].

**Table 6:** Equations of linear, Arrhenius, and Cragoe mixing rules.

Model	Equation	Source
Linear	$MFR_{mix}=x_{1}MFR_{1}+x_{2}MFR_{2}$	[38]
Arrhenius	$\ln\left(MFR_{mix}\right)=x_{1}\ln\left(MFR_{1}\right)+x_{2}\ln\left(MFR_{2}\right)$	[39]
Cragoe	$\frac{1}{\ln\left(L\;MFR_{mix}\right)}=\frac{x_{1}}{\ln\left(L\;MFR_{1}\right)}+\frac{x_{2}}{\ln\left(L\;{MFR}_{2}\right)}$	[25]

### 2.5. Model Evaluation and Error Calculation

Mixing rules are rated with three different error measures. The mean absolute error (MAE) calculates the average error per data point in one curve given in g/10 min, while the mean relative error (MRE) expresses the deviation of the calculation ($MFR_{calc}$) from the experimental value ($MFR_{exp}$) in %. The coefficient of determination $R^{2}$ gives an overall comparison of the fit and the data points where 1 is the maximum and describes a perfect prediction. $MFR_{mean}$ is the mean of the experimental data. The equations of the error measures are given in Equations (1)–(3).

(1) $$MAE=\frac{1}{N}\overset{N}{\underset{i=1}{\sum }}\left|MFR_{calc,i}-{MFR}_{exp,i}\right|$$

(2) $$MRE=\frac{1}{N}\overset{N}{\underset{i=1}{\sum }}\frac{\left|MFR_{calc,i}-{MFR}_{exp,i}\right|}{{MFR}_{exp,i}}$$

(3) $$R^{2}=1-\frac{\sum_{i=1}^{N}{\left(MFR_{exp,i}-MFR_{calc,i}\right)}^{2}}{\sum_{i=1}^{N}{\left(MFR_{exp,i}-MFR_{mean}\right)}^{2}}$$

## 3. Results

### 3.1. Adjustment of MFR with Pipe Grade Blending Partners

**Figure 1:**
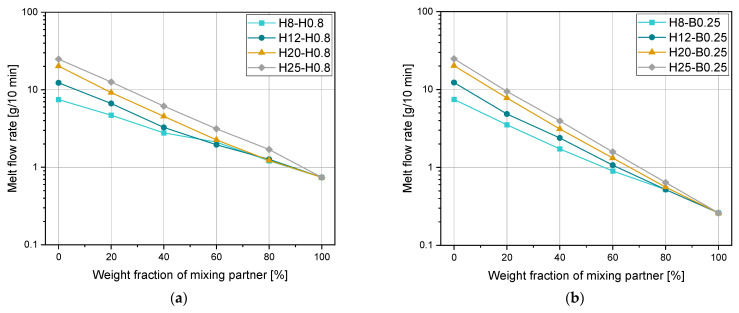
Melt flow rate depending on the weight fraction of (**a**) H0.8 and (**b**) B0.25 for H8, H12, H20, and H25.

Pipe materials are known for their very low MFR values as it is required by several standards in the pipe industry [4]. The MFR values of PP pipe grades are usually in the range of 0.25 to 4.5 g/10 min [40]. The applicability of pipe materials as blending partners to decrease the MFR was investigated by using Sample Set 1 and results are shown in Figure 1. Due to the high MFR value differences of several orders of magnitudes, the y-axis is drawn logarithmically. For both blending partners, linear dependencies on a logarithmic scale of the weight fraction were achieved for mixtures with all four initial materials. These linear relationships fit best with the equation of the Arrhenius mixing rule. However, the two blends were slightly better with the approximation of Cragoe, as indicated in bold numbers in Table 7. Values of $R^{2}$ between 0.995 and 1.000 were achieved by the mixing rule of Arrhenius, which results in reasonable deviations from the measurements of a maximum of 12%. In contrast to that, for the linear mixing rule and for the mixing rule of Cragoe, values of 0.731 to 0.899 and 0.982 to 0.988 were achieved, respectively, which corresponds to extremely high percentage deviations. For such large MFR differences, Arrhenius can be recommended for homopolymers and block copolymers as blending partners to decrease the MFR of a certain material. The influence of the chemical structure of the blending partner and thus the polymer type (i.e., homopolymer, random copolymer, or block copolymer) is evaluated in the following section.

### 3.2. Adjustment of MFR and Mechanical Properties with Extrusion Grade Blending Partners

**Figure 2:**
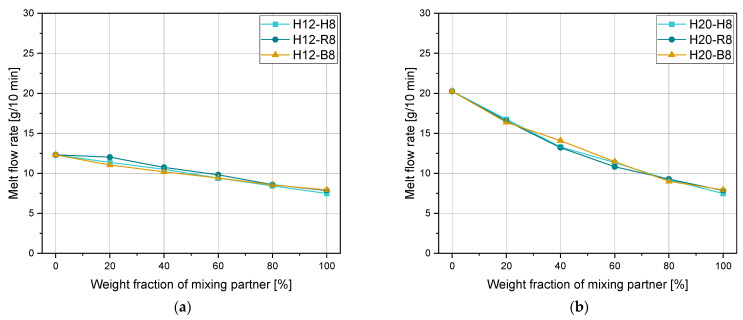

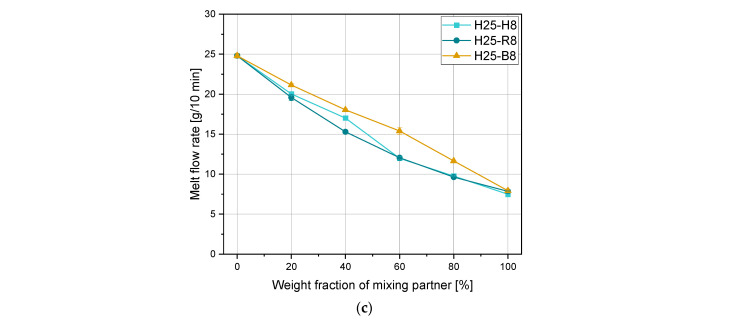
Melt flow rate depending on the weight fraction of H8, R8, and B8 of blends with (**a**) H12, (**b**) H20, and (**c**) H25.

This section deals with the simultaneous adjustment of the MFR and various mechanical properties. Therefore, three homopolymers with different MFR values in the range of 12 to 25 g/10 min were modified with a lower MFR polymer (i.e., 8 g/10 min) of different types (i.e., homopolymer, random copolymer, or block copolymer). In Figure 2, the effects of MFR adjustment with 20% blending steps are shown in a linear scale for H12, H20, and H25 blended with the extrusion materials H8, R8, and B8, respectively. The blends in Figure 2a,b show overlapping results, and thus minor differences between the different PP grades are apparent in terms of flowability. Only the H20-B8 mixture showed slightly different values at some measurement points. In contrast, Figure 2c shows a clear difference between the different blending partners. The blends with the homopolymer and the random copolymer overlap again except for 40% H25-H8. However, the blends with the block copolymer show significantly higher values and almost linear mixing behavior in a linear scale, which has been interpreted as atypical so far. As reported in [31], this behavior could be a consequence of heterophasic incorporated ethylene phases in the PP block copolymer. Furthermore, the used demolding agents could cause an unproportional increase in the MFR compared to the other samples. All calculated errors are given in Table 8. The bold number represents the best-fitting mixing rule for every single mixture. For the blends with H12, all three investigated mixing rules are sufficiently precise for the calculation. Therefore, for small MFR differences of both mixing partners, it is less critical to choose the best mixing rule for a good calculation of the MFR.

**Figure 3:**
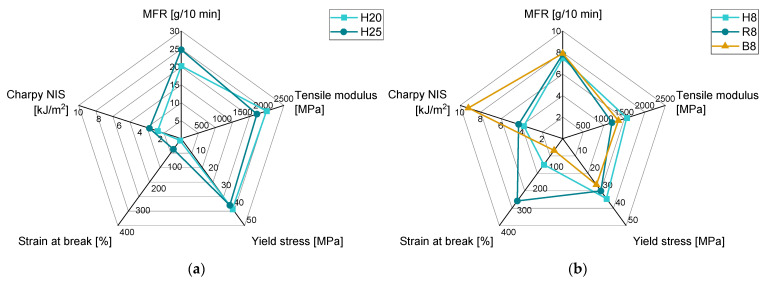
MFR and mechanical properties of (**a**) H20 and H25 and (**b**) H8, R8, and B8.

The property profiles of H20 and H25 and the blending partners H8, R8, and B8 are given in Figure 3. H20 and H25 have very similar mechanical properties with slightly more ductile properties of H25 as shown in Figure 3a. These ductile properties are here defined as lower tensile modulus, higher strain at break, and higher impact strength. The tensile moduli are approximately 2100 and 1800 MPa for H20 and H25, respectively. The yield stresses of both materials are between 38 and 40 MPa. A large difference was measured for the strain at break as values of around 8% and 50% were evaluated with the higher value corresponding to H25. The impact strengths are 2.3 kJ/m^2^ and 3.1 kJ/m^2^ for H20 and H25, respectively.

Figure 3b illustrates that the homopolymer has the highest values of tensile modulus and yield stress, while the random copolymer shows better ductility, and the block copolymer has by far the highest impact strengths. These differences in property profile are due to their molecular modification [41]. The absolute values of the tensile moduli are around 1560, 1200, and 1350 MPa, and the yield stresses are 35, 30, and 27 MPa for H8, R8, and B8, respectively. The absolute strain at break and impact strength of H8 are 120% and 3.8 kJ/m^2^. Although the strain at the break of the random copolymer is very high (290%), the impact strength is not raised with the statistical incorporation of ethylene phases (4.3 kJ/m^2^). An opposed effect is visible for the block copolymer with the lowest strain at a break of 55% and the highest impact strength of 9.2 kJ/m^2^. These general statements about the differences in property profile of different PP types are especially true for the materials listed here and presumably also for the majority of commercially available grades. However, individual material formulations may deviate from these representative property profile differences shown. The area in-between the property profiles of two different materials represent the possibilities in properties which can be achieved by blending these two materials. However, care must be taken that the change of one parameter also causes the change of the other parameters with not necessarily the same relationship and trend.

**Figure 4:**
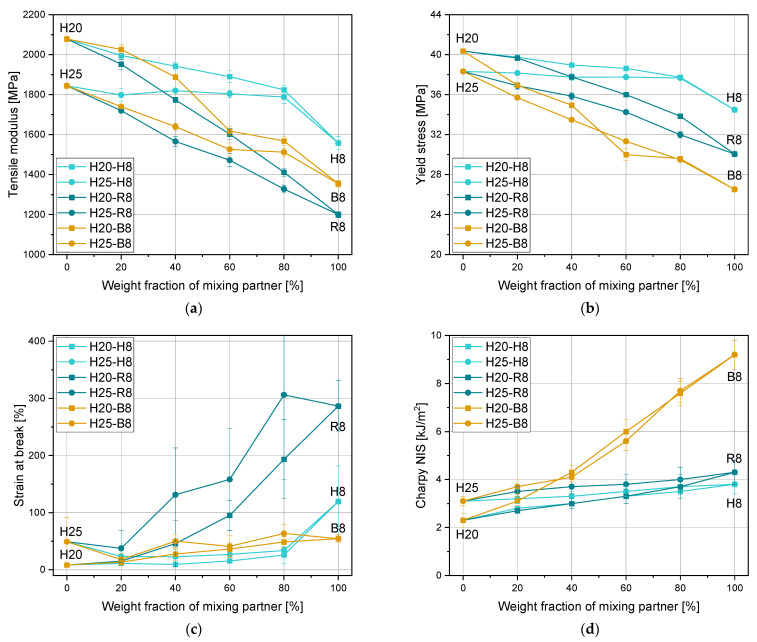
Mechanical properties (**a**) tensile modulus, (**b**) yield stress, (**c**) strain at break, and (**d**) Charpy NIS of blends with extrusion materials H8, R8, and B8.

Especially for reinforced plastics, the linear mixing rule is often used for the calculation of the tensile modulus [38]. However, in this research, it is checked if this mixing rule also works for binary blends of pure polypropylene grades. In Figure 4, the mechanical properties (a), tensile modulus, (b) yield stress, (c) strain at break, and (d) Charpy NIS from the mixtures of Sample Set 2 are shown. Tensile moduli and yield stresses show a linear decrease with an increasing amount of H8 up to 80%. The pure H8 has an even lower tensile modulus. The relationships between the tensile moduli and the yield stresses for mixtures with the copolymers as blending partners are linear, although some values deviate from the ideal linearity. The strain at break values of the blends with the homopolymer delivered constant values again up to 80%, with a more pronounced increase for the pure H8. The block copolymer revealed a slight increase for mixtures with both blending materials H20 and H25. However, for the mixtures with the random copolymer, significant increases from below 100% to almost 300% were measured. When looking at Figure 4d at the Charpy NIS, only a slight increase could be measured for mixtures with homopolymer and random copolymer blends. Nevertheless, for this property, the mixtures with the block copolymer have shown the largest influence with values of around 9 kJ/m^2^ for the pure block copolymer. Due to the inhomogeneous relationships between the mechanical properties of the material mixtures, no mixing rule is applicable. However, a prediction of the property profile trends of a material blend is possible. With the knowledge of blending partners, the prediction of property profiles becomes more accurate. The graphical preparation of the 20% increments of all material mixtures can be taken from the spider diagrams in Figures S1 to S6 in the Supplementary Materials.

### 3.3. Applicability of Property Modification on a PCR Material

**Figure 5:**
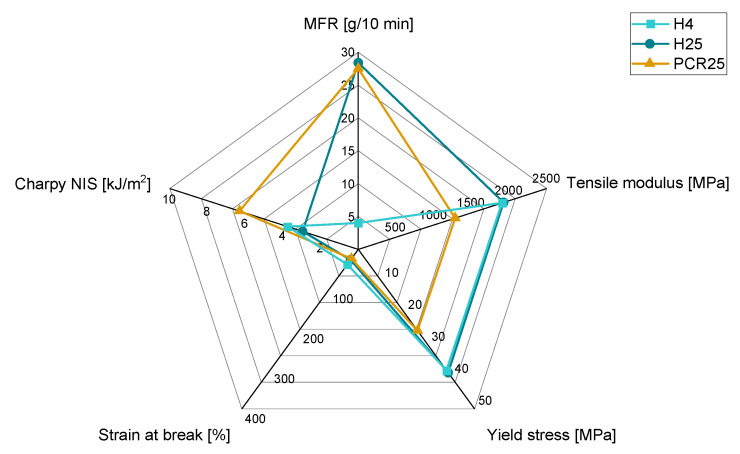
Property profiles of H25, PCR25, and H4.

As a prediction of the property profile of virgin material blends is possible up to a certain extent, in this section, the applicability for recyclates of property modification via blending with virgin materials is tested. The MFR modification was already tested in [31], which led to good applicability of the Arrhenius and Cragoe mixing rules. The overall property profiles of the blending partners H25, PCR25, and H4 are shown in Figure 5. Absolute values of H25 in Sample Set 2 and Sample Set 3 differ marginally as they were measured independently of each other, and therefore different batches were used.

H4 is used as a blending partner in this comparison of mixtures with H25 and PCR25. While H25 and PCR25 almost have the same MFR value, the mechanical properties derived from tensile tests and Charpy notched impact tests are strongly differing. The virgin material H25 has a much higher stiffness and much lower Charpy NIS compared to PCR25. The strain at break is almost the same due to macroscopic defects in PCR25 which arise due to insufficient sorting and filtering in the recycling process. These contaminations act as crack initiators and lead to stress concentrations in the material [7]. The mechanical properties of H4 are similar to H25, although the flowability is not comparable.

**Figure 6:**
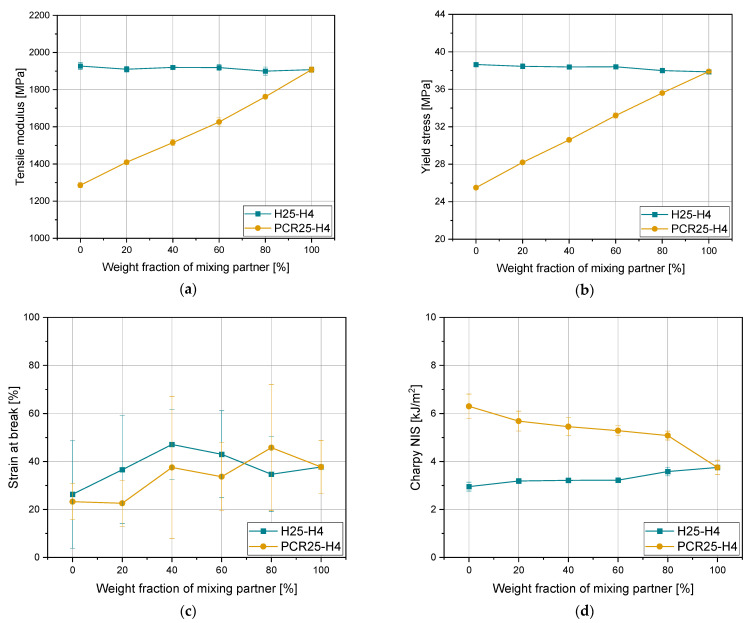
Mechanical properties (**a**) tensile modulus, (**b**) yield stress, (**c**) strain at break, and (**d**) Charpy NIS of blends with H25 and PCR25.

In Figure 6, the modification of mechanical properties of H25 and PCR25 when mixing with H4 is shown. As there is almost no difference between H25 and H4 in terms of stiffness, an almost constant value of around 1900 MPa and 38 MPa for tensile modulus and yield stress, respectively, is shown in Figure 6a,b. The recyclate PCR25 initially has much lower pre-yield values of around 1300 MPa and 25 MPa for tensile modulus and yield stress, respectively. This allows for a much better property modification with a virgin homopolymer. A linear dependence of both properties with a $R^{2}$ of 0.99 could be derived. The similarity of strain at break values of all three materials led to only marginal changes with high standard deviations for pure materials and blends. The initial impact strength value of H25 is much lower than that of PCR25. The value of H4 is in-between those two materials. While in H25-H4, the value changes in a linear way depending on the mixing ratio, in PCR25-H4, the recyclate dominates the result of the mixture up to a share of 80% H4. Between 80% and 100%, a significant drop in the value occurs. This might come from macroscopic particles which are remaining in the recyclate, although the notch of the specimen ensures a targeted break. Therefore, it can be assumed, the cleaner the recyclates the better the modification of the impact strength by mixing with other materials. The corresponding spider diagrams are again shown in the Supplementary Materials as Figures S7 and S8, which show the possibility to change the MFR of a material with almost no change of the mechanical properties (H25-H4) and the adaptability of modifying the flowability and the mechanical properties of a post-consumer recyclate (PCR25-H4).

## 4. Discussion

The flowability of a material can easily be adjusted by blending with a material partner with a different MFR. While it was already tested to change the MFR of a broad range of materials with a homopolymer with an MFR of 4 g/10 min as a blending partner [31], for this work, the MFR range of the blending partner was extended to both directions. On the one hand, pipe materials (PP-H and PP-B) with a minimum MFR of 0.25 g/10 min, and on the other hand, extrusion materials (PP-H, PP-R, and PP-B) with a constant MFR of 8 g/10 min were tested. For the blends with pipe materials, it was shown that the MFR changed significantly by adding only small amounts of the blending partner. The MFR changes can be calculated by the mixing rule of Arrhenius. The different extrusion materials did not show such a clear trend as homopolymers and random copolymers have shown a logarithmic decrease in the MFR following the Arrhenius and Cragoe mixing rules, while blending with block copolymers showed a pronounced linear decrease in MFR. The higher values of the PP-B compared to PP-H and PP-R could be caused by the demolding additives in the used grade. Furthermore, blends with a low MFR difference (e.g., H12-H8, H12-R8, and H12-B8) can also be well approximated with the linear mixing rule.

Mechanical properties of virgin materials can easily be modified by blending with other polymers or additives such as fibers and fillers [42,43,44,45], although the latter worsens the recyclability. This study revealed a rather minor change when blending a homopolymer with another homopolymer. While the values of tensile modulus and yield stress dropped, the strain at break values and impact strength values increased slightly. However, the property adjustments are strongly dependent on the grade which is used for blending. Copolymers as blending partners led to a significantly larger property adjustment. The random copolymer decreased the tensile modulus and the yield stress significantly. In contrast, the strain at break and impact strength values increased with a major change in the ductility. Block copolymers mainly led to a significant drop in tensile modulus and yield stress and a huge increase in impact strength. Table 9 contains a summary of these statements. In numbers, the modulus was decreased by a maximum of 42% and the yield stress by 34%. A 23-fold and 4-fold increase was obtained for strain at break and Charpy NIS, respectively. Furthermore, the initial composition of the plastics and recyclates has to be taken into account as several additives and fillers are known to change the material properties [46]. Nevertheless, this exceeds the scope of this research. The exact compositions of the recyclates are usually unknown, which makes it complicated to create models with all components.

In general, it is very well possible to adjust the flow properties and mechanical properties of recyclates simultaneously by blending. Tensile modulus and yield stress, i.e., parameters that are characteristic of pre-yield, can be calculated using the linear mixing rule, as is also accomplished for virgin materials. Post-yield properties such as strain at break are strongly dependent on the purity and quality of the recyclates. Macroscopic influences lead to strongly weakened results [7]. The strain at break values in this study are too similar to draw a final conclusion. The notched impact strength is less linear for recyclates than for virgin materials since the recyclates and their contaminants are dominant.

The targeted property adjustment of recyclates by blending with virgin materials opens many areas of application that were previously not possible due to the initial properties of recyclates. Nevertheless, the long-term behavior of recycled materials must not be ignored, as there are still considerable deficits in this area [3,4,5]. Depending on the intended property profile and whether the recyclate or the virgin material should be the main component, the right blending ratio must be selected.

## 5. Conclusions

The aim of this paper is to show possibilities for the simultaneous modification of material properties which are relevant for processing and the final application. Selected materials for modification were limited to circular economy-compliant components; therefore, only commercially available polypropylene grades were used. For various polypropylene blends consisting of blending partners from different virgin and recycled materials, MFR measurements, tensile tests, and Charpy notched impact tests were conducted. Using virgin pipe grades to decrease the MFR is a promising approach to maintaining a high amount of recyclate for a certain application. Additionally, decreasing the MFR with different PP types, namely homopolymers, random copolymers, and block copolymers, is feasible with simultaneous adjustment of the mechanical properties since each type offers an individual property profile. Nevertheless, block copolymers with demolding agents behave differently with respect to MFR adaption. All material modifications tested on virgin PP blends are also applicable to high-quality recyclates.

Property profile modification of recyclates due to compounding with virgin materials opens the possibility of incorporation of recyclate for various application areas. As a modification, using a material with a known property profile is very well feasible for both flowability and mechanical properties and thus the spectrum of application increases drastically. If a high sorting depth is ensured and therefore a pure recyclate is provided, the resulting recyclates can further be used for demanding applications. Therefore, the provided trends can be used for predicting the properties of polypropylene blends and calculating the required amount of the mixing partner to achieve a targeted property profile. Nevertheless, for industrial applications, the models might not be applicable if unknown components such as fillers and additives are present in the recyclate. In the future, adjustments of further properties such as color and odor can be targeted to close the loop toward a circular economy of plastics.

## Figures and Tables

**Table 2:** List of used material grades with their corresponding designation; MFR values are given in g/10 min.

Designation	Material	MFR	Designation	Material	MFR
H0.8	HA507MO	0.8	H25	HG385MO	25
H4	HC205TF	4	R8	RD204CF	8
H8	HD204CF	8	B0.25	BA202E	0.25
H12	HE125MO	12	B8	BD310MO	8
H20	HF955MO	20	PCR25	Purpolen PP	25

**Table 3:** Description of sample sets and blending partner composition.

Set	Blend	H0.8	H4	H8	H12	H20	H25	R8	B0.25	B8	PCR25
1	H8-H0.8	X	-	X	-	-	-	-	-	-	-
	H12-H0.8	X	-	-	X	-	-	-	-	-	-
	H20-H0.8	X	-	-	-	X	-	-	-	-	-
	H25-H0.8	X	-	-	-	-	X	-	-	-	-
	H8-B0.25	-	-	X	-	-	-	-	X	-	-
	H12-B0.25	-	-	-	X	-	-	-	X	-	-
	H20-B0.25	-	-	-	-	X	-	-	X	-	-
	H25-B0.25	-	-	-	-	-	X	-	X	-	-
2	H12-H8	-	-	X	X	-	-	-	-	-	-
	H20-H8	-	-	X	-	X	-	-	-	-	-
	H25-H8	-	-	X	-	-	X	-	-	-	-
	H12-R8	-	-	-	X	-	-	X	-	-	-
	H20-R8	-	-	-	-	X	-	X	-	-	-
	H25-R8	-	-	-	-	-	X	X	-	-	-
	H12-B8	-	-	-	X	-	-	-	-	X	-
	H20-B8	-	-	-	-	X	-	-	-	X	-
	H25-B8	-	-	-	-	-	X	-	-	X	-
3	H25-H4	-	X	-	-	-	X	-	-	-	-
	PCR25-H4	-	X	-	-	-	-	-	-	-	X

**Table 4:** Weight fractions $x_{1}$ and $x_{2}$ for each material blend.

Mixture	1	2	3	4	5	6
$x_{1}$	100	80	60	40	20	0
$x_{2}$	0	20	40	60	80	100

**Table 5:** Processing parameters of the compounding step.

Temperatures	
Filling zone	30 °C
Zone 2	170 °C
Zone 3	195 °C
Zone 4–12	210 °C
Die	210 °C
Processing parameters	
Rotational speed	400 rpm
Throughput	8 kg/h

**Table 7:** MAE (in g/10 min), MRE (in %) and $R^{2}$ for pipe material blends.

Blend	Linear			Arrhenius			Cragoe		
	MAE	MRE	R^2^	MAE	MRE	R^2^	MAE	MRE	R^2^
H8-H0.8	0.94	39.66	0.899	**0.08**	**3.54**	**0.997**	0.23	9.04	0.992
H12-H0.8	2.16	83.46	0.826	0.24	7.78	0.996	**0.17**	**4.76**	**0.997**
H20-H0.8	4.13	134.58	0.784	0.47	12.01	0.995	**0.26**	**5.13**	**0.998**
H25-H0.8	4.59	110.40	0.823	**0.12**	**3.25**	**1.000**	1.04	18.48	0.984
H8-B0.25	1.47	119.06	0.796	**0.10**	**5.70**	**0.998**	0.20	12.93	0.993
H12-B0.25	2.72	184.29	0.750	**0.21**	**8.21**	**0.995**	0.31	14.67	0.994
H20-B0.25	4.71	264.58	0.731	**0.22**	**7.67**	**0.999**	0.65	19.33	0.987
H25-B0.25	5.75	272.42	0.733	**0.10**	**1.61**	**1.000**	0.97	24.72	0.982

**Table 8:** MAE (in g/10 min), MRE (in %) and $R^{2}$ for extrusion material blends.

Blend	Linear			Arrhenius			Cragoe		
	MAE	MRE	R^2^	MAE	MRE	R^2^	MAE	MRE	R^2^
H12-H8	**0.03**	**0.28**	**0.999**	0.18	1.82	0.992	0.21	2.14	0.989
H20-H8	0.81	6.60	0.977	**0.12**	**0.96**	**0.999**	0.19	1.49	0.998
H25-H8	0.97	7.37	0.981	**0.42**	**2.61**	**0.990**	0.64	4.09	0.984
H12-R8	**0.19**	**1.77**	**0.983**	0.27	2.49	0.972	0.30	2.75	0.968
H20-R8	1.06	8.80	0.962	0.28	2.39	0.996	**0.15**	**1.25**	**0.998**
H25-R8	1.46	10.90	0.965	0.19	1.47	0.999	**0.08**	**0.50**	**1.000**
H12-B8	0.22	2.19	0.988	0.09	0.86	0.997	**0.06**	**0.62**	**0.998**
H20-B8	0.90	7.48	0.977	0.20	1.75	0.997	**0.20**	**1.74**	**0.996**
H25-B8	**0.23**	**1.51**	**0.997**	1.39	8.82	0.965	1.63	10.26	0.953

**Table 9:** Influence of PP type on the overall property profile for blending with a high MFR homopolymer (direction of arrows indicate increasing or decreasing values and number of arrows indicate the extent of the change).

Blending Partner	MFR	Tensile Modulus	Yield Stress	Strain at Break	Charpy NIS
	g/10 min	MPa	MPa	%	kJ/m^2^
Homopolymer H8	↓	↓	↓	↑	↑
Random copolymer R8	↓	↓↓↓	↓↓	↑↑	↑↑
Block copolymer B8	↓	↓↓	↓↓↓	-	↑↑↑

## Data Availability

The data presented in this study are available on request from the corresponding author.

## Supplementary Materials

The following supporting information can be downloaded at https://www.mdpi.com/article/10.3390/polym15071717/s1. Figure S1: Property profiles of all blends of H20-H8, Figure S2: Property profiles of all blends of H25-H8; Figure S3: Property profiles of all blends of H20-R8; Figure S4: Property profiles of all blends of H25-R8; Figure S5: Property profiles of all blends of H20-B8; Figure S6: Property profiles of all blends of H25-B8; Figure S7: Property profiles of all blends of H25-H4; Figure S8: Property profiles of all blends of PCR25-H4.
