# Supplementary material for: Simultaneous Modification of Properties Relevant to the Processing and Application of Virgin and Post-Consumer Polypropylene
Source: Polymers (Basel). 2023 Mar 30;15(7):1717. doi: 10.3390/polym15071717 (PMC10097265; doi:10.3390/polym15071717)
Supplement: Supplementary file 1 [file polymers-15-01717-s001.zip › polymers-2236639-supplementary.pdf]

polymers

MDPI

Supplementary Material

Simultaneous Modification of Properties Relevant to the Processing and Application of Virgin and Post-Consumer Polypropylene

Ines Traxler [1,*], Hannes Kaineder [2] and Joerg Fischer [2]

[1] Competence Center CHASE GmbH, Altenberger Strasse 69, 4040 Linz, Austria
[2] Institute of Polymeric Materials and Testing, Johannes Kepler University, Altenberger Strasse 69, 4040 Linz, Austria
* Correspondence: ines.traxler@chasecenter.at; Tel.: +43-664-8568524

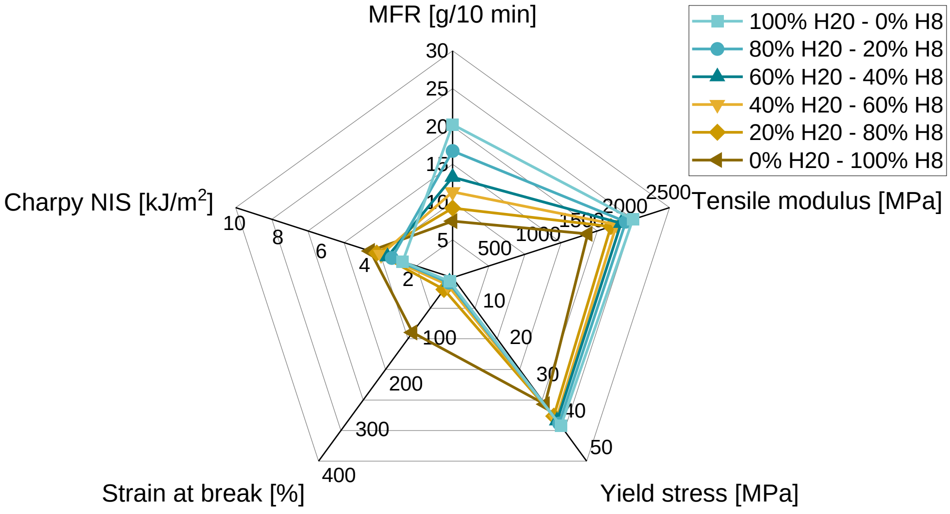

Figure S1. Property profiles of all blends of H20-H8.

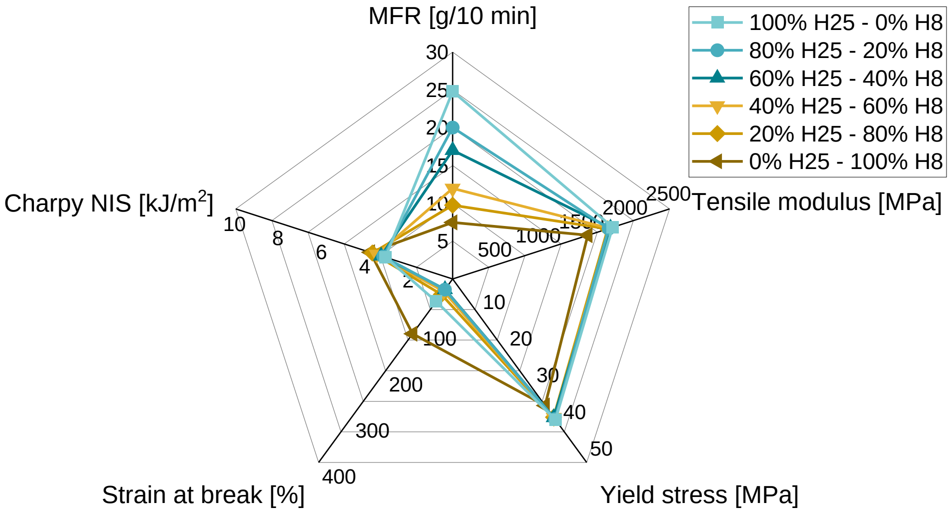

Figure S2. Property profiles of all blends of H25-H8.

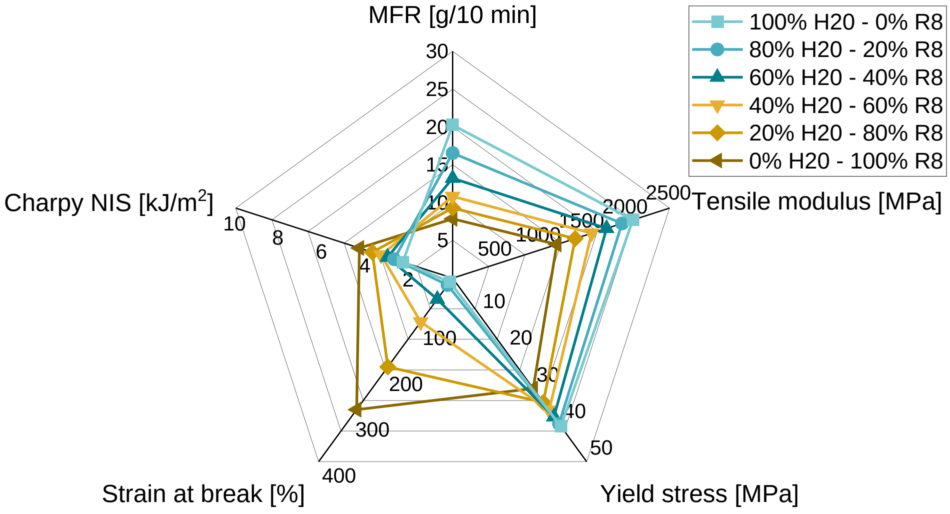

Figure S3. Property profiles of all blends of H20-R8.

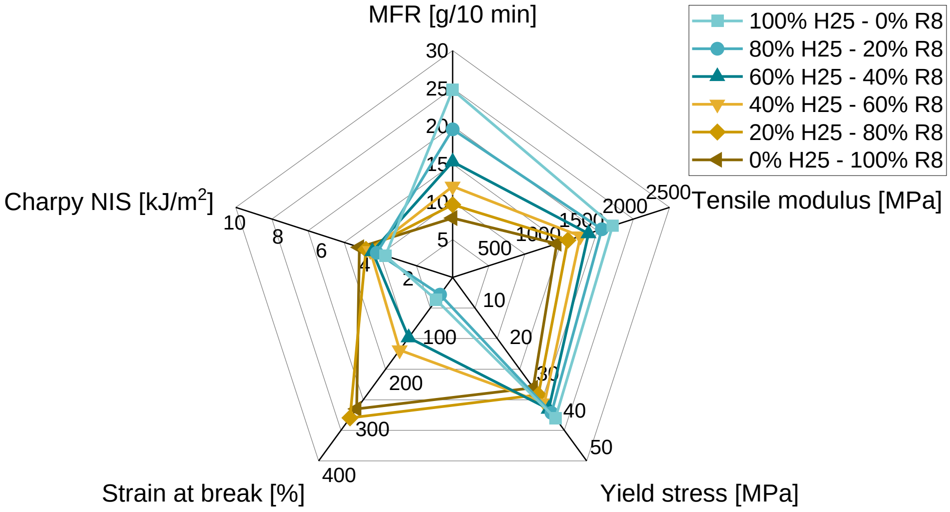

Figure S4. Property profiles of all blends of H25-R8.

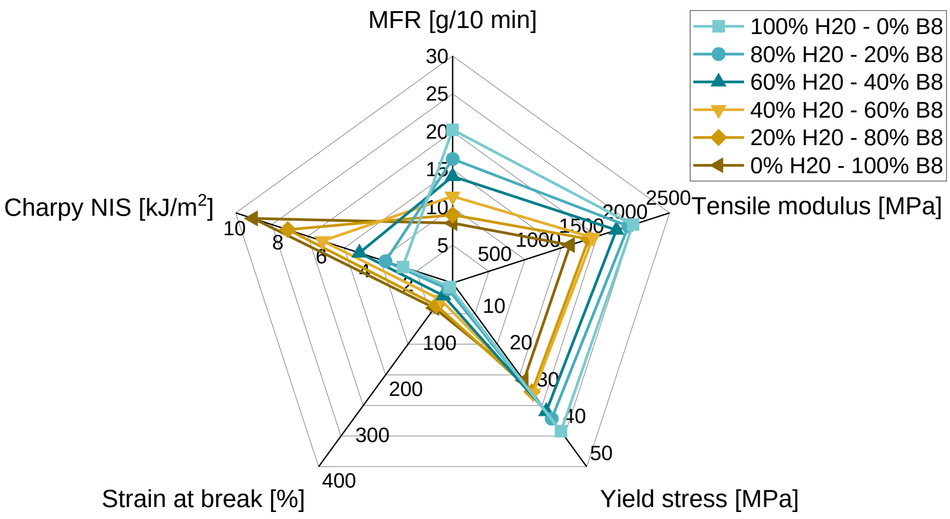

Figure S5. Property profiles of all blends of H20-B8.

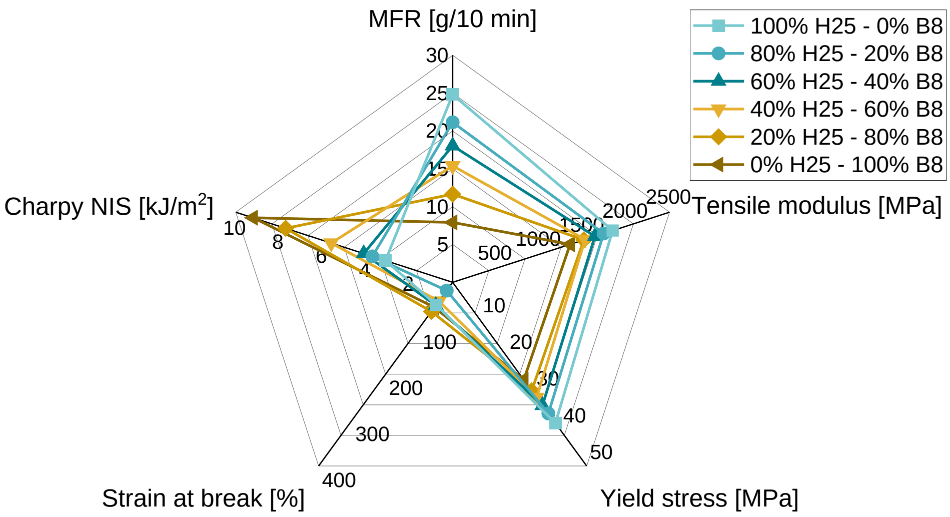

Figure S6. Property profiles of all blends of H25-B8.

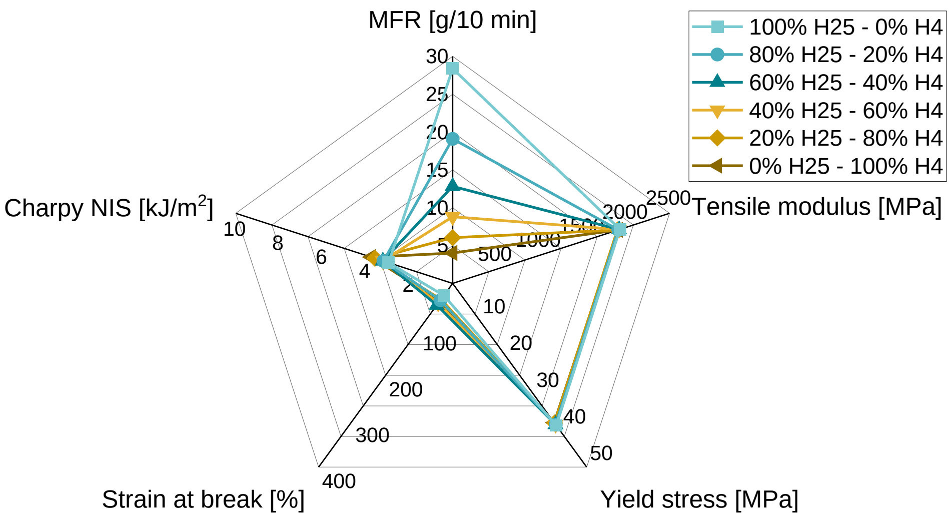

Figure S7. Property profiles of all blends of H25-H4.

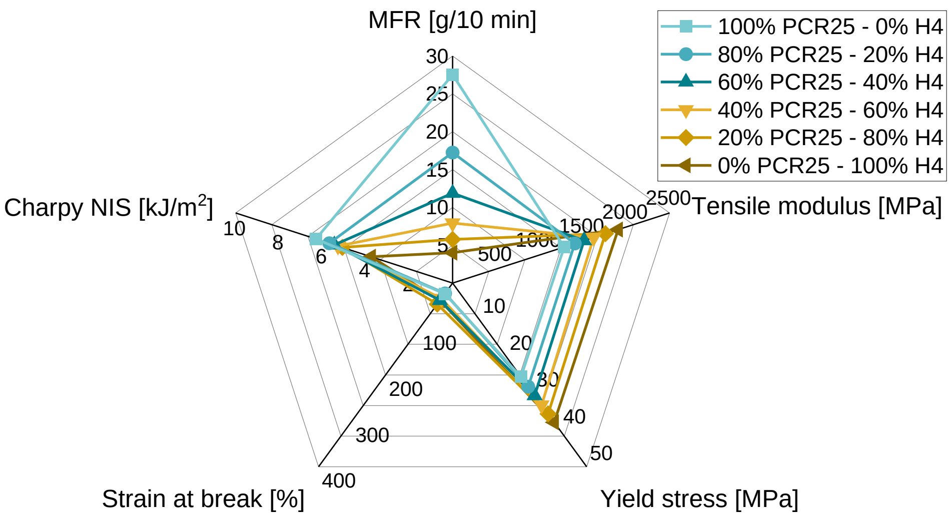

Figure S8. Property profiles of all blends of PCR25-H4.
